# A Case of Proteus Syndrome, Suspected as Maffucci Syndrome in a Chinese Child

**DOI:** 10.1155/crpe/9848886

**Published:** 2025-08-22

**Authors:** Lin Juan, Liang Jing, Tang Ben-Yu, Li Yin-Ya, Chen Dan-Chun, Zhu Shun-Ye

**Affiliations:** ^1^Department of Pediatrics, The Third Affiliated Hospital, Sun Yat-sen University, Guangzhou 510630, China; ^2^Department of Pathology, The Third Affiliated Hospital, Sun Yat-sen University, Guangzhou 510630, China

**Keywords:** AKT1, case report, Maffucci syndrome (MS), overgrowth, Proteus syndrome (PS)

## Abstract

Proteus syndrome (PS) is an exceptionally rare disorder characterized by asymmetric and disproportionate overgrowth of connective tissues. We report the case of an 8-year-old female presenting with irregular cranial protrusion, bilateral supraorbital ridge enlargement, overgrowth of the right hand and left foot, and a pelvic MRI revealing an ovarian tumor. Initially, the patient was suspected of having Maffucci syndrome (MS) upon admission. Genetic analysis of a lesion sample from the fifth toe of the left foot identified a heterozygous point mutation, 49G > A (p.Glu17Lys), in the AKT1 gene. The patient met the clinical-molecular diagnostic criteria established by Leslie G. Biesecker, scoring 15 points, thereby confirming the diagnosis of AKT1-related PS. This case report contributes to the enhanced understanding of PS diagnosis associated with AKT1 mutations.

## 1. Introduction

Proteus syndrome (PS), a rare congenital disorder characterized by sporadic occurrence, was first identified and described by Cohen and Hayden in 1979 [1]. Epidemiological studies estimate the prevalence of PS to be less than 1 per 1,000,000 live births, with a reported male-to-female ratio of 1.9:1.5 [2–4]. The clinical presentation of this dysplastic syndrome exhibits considerable heterogeneity and lacks pathognomonic features, often leading to diagnostic challenges and potential misdiagnosis. In this report, we present a case of PS associated with an AKT1 mutation, characterized by a distinctive facial phenotype accompanied by swelling of both hands and feet, which was diagnosed at our institution.

## 2. Case Presentation

An 8-year-old female patient presented with a progressively enlarging growth involving the skull, fingers, and toes. She was delivered via cesarean section at 39 weeks of gestation due to fetal decelerations, with a birth weight of 3.65 kg, to a 35-year-old G2P2 mother. No congenital abnormalities were noted at birth. However, at the age of 2 years, she developed cranial overgrowth, which gradually progressed to involve the right hand and left foot. These manifestations remained untreated. The progressive enlargement of the lesions resulted in functional impairment, including the inability to write with the right hand and difficulty finding appropriately sized footwear. In addition, the patient exhibited varicose veins in the left leg. Born and raised in South America, she relocated to China at the age of 3. Family history was noncontributory, and routine prenatal examinations during the mother's pregnancy revealed no abnormalities, with no prenatal diagnostic procedures performed.

**Figure 1:**
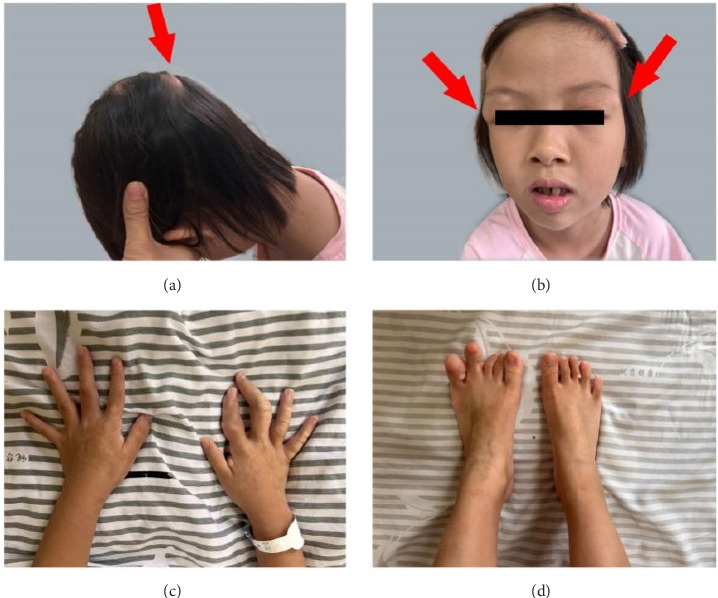
Representative images of the patient. (a) Prominent occipital skull protrusions (indicated by arrow). (b) Bilateral supraorbital ridge prominence (marked by arrow). (c) Asymmetric overgrowth involving the right thumb and middle finger. (d) Overgrowth affecting the fourth and fifth digits of the left foot.

Physical examination revealed the following anthropometric measurements: height 130 cm (+0.1 SD) and weight 23.8 kg (−0.2 SD). The patient presented with facial dysmorphism characterized by elongation and bilateral ptosis. Ocular, thyroid, and cardiorespiratory systems were unremarkable upon clinical evaluation. No palpable lymphadenopathy, abdominal ascites, cutaneous café-au-lait spots, or dermatological abnormalities were detected. Bilateral lower extremities demonstrated symmetrical length. Cranial examination identified prominent occipital protuberances and supraorbital arching. Podiatric assessment revealed multiple nodular lesions on the fourth and fifth digits of the left foot. Orthopedic evaluation of the right hand demonstrated significant swelling and deformity of the thumb and middle finger metacarpophalangeal joints, resulting in complete loss of flexion (Figures 1(a), 1(b), 1(c), and 1(d)).

**Figure 2:**
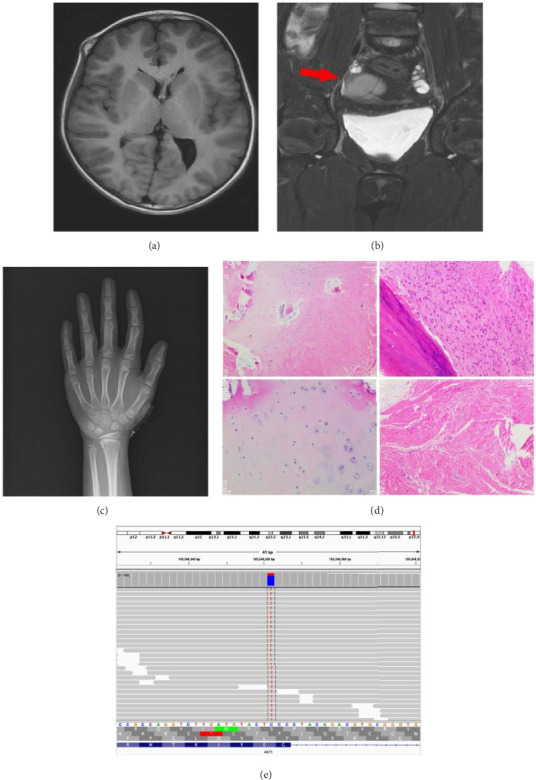
AKT1-related PS. (a) Cranial magnetic resonance imaging (MRI) findings indicative of congenital developmental anomalies. (b) Pelvic MRI demonstrating an ovarian-derived neoplasm. (c) Radiographic imaging of the left hand. (d) Histopathological alterations in osseous lesions obtained from the right thumb and left fifth digit. (e) Next-generation sequencing (NGS) analysis revealing a heterozygous missense mutation in exon 4 of the AKT serine/threonine kinase 1 (AKT1) gene (c.49G > A, p.Glu17Lys).

Auxiliary diagnostic investigations revealed normal findings in electrolyte levels, complete blood count, urinalysis, liver function tests, bone metabolism markers, and serum thyroid-stimulating hormone (TSH). Serum tumor marker concentrations were within normal reference ranges as were growth hormone (GH) and insulin-like growth factor-1 (IGF-1) levels. Sex hormone profiles were unremarkable. Ultrasonographic evaluation identified multiple superficial masses localized to the left plantar region, right finger joints, and bilateral frontal cranial areas. Doppler ultrasonography of the lower extremities demonstrated bilateral great saphenous vein dilatation with tortuous expansion of superficial venous structures. Echocardiographic assessment revealed interventricular septal hypertrophy with preserved left ventricular systolic function. Cranial magnetic resonance imaging (MRI) demonstrated volumetric reduction of the right cerebral hemisphere, brainstem, cerebellum, lateral ventricle, and corpus callosum compared with contralateral structures, accompanied by mild midline shift to the left, suggestive of congenital developmental anomalies. Bilateral parietal bone enlargement and protrusions of the right frontal bone and lateral orbit were consistent with osteofibrous dysplasia (Figure 2(a)). Paranasal sinus computed tomography (CT) revealed irregular osseous fragments within the soft tissue inferior to the left temporomandibular joint, indicative of abnormal ossification. Multiple osseous protrusions were observed in the inferolateral aspect of the right zygomatic bone, right frontal bone, and right anterior circumvertebral tubercle, consistent with developmental abnormalities. Nasopharyngeal soft tissue thickening was suggestive of adenoid hypertrophy. Pelvic MRI identified a well-defined, thick-walled cystic lesion (4.7 × 3.4 cm) with irregular margins posterior to the bladder (Figure 2(b)). Spinal radiography demonstrated mild scoliosis with an anomalous vertebral count of 11 thoracic vertebrae. While bone age corresponded to chronological age, radioulnar joint abnormalities were noted, characterized by relative radial elongation compared with the ulna (Figure 2(c)). Lymphadenopathy was absent. Ophthalmological examination revealed no abnormalities in fundoscopic findings, visual acuity, or visual fields.

Biopsy specimens were obtained from the right thumb and the fifth toe of the left foot, revealing osteochondroma and fibroma, respectively, both of which were identified as benign lesions (Figure 2(d)). Based on these findings, a preliminary diagnosis of Maffucci syndrome (MS) was considered. However, immunohistochemical analysis of the pathological tissues demonstrated negative staining for isocitrate dehydrogenase 1 (IDH-1), which did not support the diagnosis of MS. Whole exome sequencing (WES) performed on peripheral blood genomic DNA yielded normal results. Given the suspicion of a somatic cell gene mutation, DNA was subsequently extracted from the soft tissue mass of the fifth toe of the left foot and subjected to capture-based targeted sequencing analysis using a panel encompassing 520 cancer-related genes (Burning Rock Biotech, Guangzhou, China). Genetic testing identified a heterozygous point mutation, 49G > A (p.Glu17Lys), in the exon region of the AKT1 gene, with an allele frequency of 21.57% (Figure 2(e)). According to the OMIM database, somatic mutations in the AKT1 gene have been associated with various diseases, including breast cancer, colorectal cancer, ovarian cancer, and PS. Based on the clinical characteristics and auxiliary examination findings of this case, the most probable diagnosis was determined to be PS.

## 3. Discussion

PS presents with heterogeneous clinical manifestations that significantly impair patients' quality of life. The diagnostic process is challenging due to phenotypic variability and nonspecific imaging features. In this case, the initial differential diagnosis included MS, which is characterized by multiple enchondromas and benign cartilaginous lesions in the appendicular skeleton, often accompanied by multiple hemangiomas and/or lymphangiomas [5]. Recent molecular studies have identified IDH1 and IDH2 gene mutations in both enchondromas and spindle cell hemangiomas associated with MS [6]. However, pathological immunohistochemical analysis and genetic testing in this case excluded MS. The molecular pathogenesis of PS involves somatic activating mutations in the AKT1 gene [7]. This case fulfilled the clinical-molecular diagnostic criteria established by Biesecker and Sapp [8], demonstrating a score of 15 points with the identification of a heterozygous point mutation (49G > A, p.Glu17Lys) in the AKT1 gene, thereby confirming the diagnosis of AKT1-related PS. Consequently, the final diagnosis was revised to PS.

In the differential diagnosis of PS, it is imperative to consider other syndromes exhibiting overlapping clinical features but distinct genetic etiologies, including congenital lipomatous overgrowth, vascular malformations, epidermal nevi, scoliosis/skeletal and spinal (CLOVES) syndrome, and PTEN hamartoma tumor syndrome (PHTS) [9]. CLOVES syndrome, classified within the PIK3CA-related overgrowth spectrum (PROS), typically manifests clinically within the first year of life, with progressive development of additional symptoms in subsequent stages [10]. PHTS, characterized by vascular anomalies in over 50% of affected individuals and adipose tissue overgrowth, may be misdiagnosed as PROS disorders, including CLOVES syndrome or fibroadipose vascular anomaly (FAVA) [11].

The management of PS necessitates a multidisciplinary approach, with surgical corrective interventions typically constituting the primary therapeutic strategy [2]. In the present case, pelvic MRI identified an ovarian-origin tumor, with ovarian granulosa cell tumor remaining a differential diagnosis following consultation with gynecological specialists, who recommended surgical resection. Concurrently, orthopedic specialists advised amputation of the hand and foot malformations, consistent with previous studies documenting significant ossification defects in the affected foot tissue of PS patients [12, 13]. Following multidisciplinary case discussion, pulmonary vascular CT was proposed to exclude thrombosis and pulmonary bullae; however, the patient declined further diagnostic evaluation and treatment. From a molecular perspective, AKT1, a serine-threonine kinase within the AKT/PI3K/mTOR pathway, plays a crucial role in apoptosis inhibition and growth promotion [14]. While miransetib, an oral allosteric pan-AKT inhibitor targeting both active and inactive AKT forms [15], represents a potential therapeutic option, its unavailability in China limits its clinical application. Alternatively, rapamycin (sirolimus), a specific mTOR inhibitor with established antitumor properties, has been reported in the management of PS, as demonstrated by Deborah J. Marsh's case study of a 2-year-old PS patient [16]. In the Chinese clinical context, rapamycin remains an off-label treatment for PS, necessitating formal approval from the Medical Ethics Committee prior to administration.

## 4. Conclusion

PS is characterized by intricate clinical manifestations, marked phenotypic heterogeneity, and significant variability in disease presentation. Tissue-based genetic analysis has emerged as a critical diagnostic tool in the identification and characterization of this disorder.

## Data Availability

The data that support the findings of this study are available from the corresponding author upon reasonable request. The data are not publicly available due to privacy or ethical restrictions.
